# Live Longer with Vitamin D?

**DOI:** 10.3390/nu7031871

**Published:** 2015-03-12

**Authors:** Uwe Gröber, Jörg Reichrath, Michael F. Holick

**Affiliations:** 1 Academy for Micronutrient Medicine, Zweigertstr, 55, 45130 Essen, Germany; 2 Department of Dermatology, The Saarland University Hospital, 66421 Homburg/Saar, Germany; E-Mail: Joerg.Reichrath@uniklinikum-saarland.de; 3 Boston University Medical Center, 85 East Newton Street M-1013, Boston, MA 02118, USA; E-Mail: mfholick@bu.edu

**Keywords:** vitamin D, 25-hydroxyvitamin D, vitamin D deficiency, breast cancer, cancer, overall mortality

## Abstract

The global burden of vitamin D deficiency or insufficiency is of great concern for public health. According to recent studies, vitamin D deficiency is an important etiological factor in the pathogenesis of many chronic diseases. Whether or not there is a connection between 25-hydoxyvitamin D (25(OH)D) status and overall mortality is a matter of considerable debate. A new meta-analysis confirmed that low 25(OH)D levels were associated with a significant increased risk for all-cause mortality. Individuals with severe vitamin D deficiency have almost twice the mortality rate as those with 25(OH)D level ≥ 30 ng/mL, (≥75 nmol/L). Unlike previous meta-analyses which suggested that serum 25(OH)D > 50 ng/mL was associated with increased mortality, this new analysis found that there was no increased risk even when 25(OH)D levels were ≥70 ng/mL. In general, closer attention should be paid to vitamin D deficiency in medical and pharmaceutical practice than has been the case hitherto. The results of these studies are consistent with the recommendation to improve the general vitamin D status in children and adults by means of a healthy approach to sunlight exposure, consumption of foods containing vitamin D and supplementation with vitamin D preparations.

## 1. Introduction

Vitamin D deficiency and insufficiency is a major health concern worldwide. Vitamin D deficiency (25(OH)D < 20 ng/mL) and insufficiency (25(OH)D: 20–29 ng/mL) has been associated with many chronic illnesses [1,2,3]. These include skeletal diseases such as rickets, osteomalacia, osteoporosis and nonskeletal diseases including autoimmune diseases (e.g., multiple sclerosis, type 1 diabetes), inflammatory bowel disease (e.g., Crohn’s disease), infections (such as infections of the upper respiratory tract), immune deficiency, cardiovascular diseases (e.g., coronary heart disease, hypertension, heart failure, sudden cardiac death), cancer (e.g., colon cancer, breast cancer, non-Hodgkin’s lymphoma) and neurocognitive disorders (e.g., Alzheimer disease) [1,2,3,4,5,6,7]. Meta-analyses have suggested that both low and higher 25(OH)D levels are associated with increased risk of mortality [8,9,10]. UV radiation may affect many processes in the human body independent of vitamin D production. Solar UV exposure may account for the benefits with higher 25(OH)D serum levels. Thus, 25(OH)D concentration may be a measure of sun exposure. However, it is very difficult or even impossible, to understand which of the processes are mediated by UV alone and which via vitamin D [11]. Whether or not there is a connection between vitamin D status and overall mortality is a matter of considerable debate [12,13,14,15,16].

## 2. Vitamin D and Mortality

In December 2013 Zheng *et al.* [17] reported on a meta-analysis of 42 randomized controlled trials that evaluated short and long-term vitamin D supplementation on overall mortality. They showed that in 29 studies, short-term (less than three years) vitamin D therapy did not reduce all-cause mortality. However, sub-analysis of the 13 randomized and placebo-controlled studies that were conducted for three years or longer, revealed that vitamin D supplementation reduced all-cause mortality by a significant 6% (RR 0.94; 95% CI = 0.90 to 0.98; *p* = 0.001). Evaluation of these studies indicated that long-term vitamin D supplementation (e.g., 800 IU per day) is effective in reducing overall mortality, but not if vitamin D is taken for less than three years [17]. In addition to the effect of the duration of vitamin D supplementation on reducing mortality risk Chowdhury *et al.* [18] reported that the dose of vitamin D, the change in serum 25(OH)D and the form of vitamin D also can have a significant influence on risk of cause for death. Their systematic review and meta-analysis of 73 observational cohort and 22 randomised controlled trials with 849,412 and 30,716 participants respectively revealed in the observational cohort studies, comparing bottom *vs.* top thirds of baseline 25(OH)D serum level (25(OH)D ≥ 30 v < 10 ng/mL) a pooled relative risks of 1.14 for death from cancer, 1.30 for other non-vascular, non-cancer death, 1.35 for death from cardiovascular disease and 1.35 for all-cause mortality. Additional analyses by various cut-off values of 25(OH)D serum levels showed a significant inverse association with all-cause mortality (*p* < 0.05). Each decline of 25(OH)D by 10 ng/mL was associated with a 16% increased risk of all-cause mortality. In the randomized controlled trials where vitamin D2 (dose range: 208–4500 IU/day) or vitamin D3 (dose range: 10–6000 IU/day) were given alone *vs.* placebo or no treatment, vitamin D3 significantly reduced the mortality by 11%, whereas vitamin D2 increase the mortality by 4%. The increased risks of mortality for vitamin D2 were observed in studies with lower intervention doses (≤600 IU/day) and shorter average intervention periods (<1.5 years) [18].

**Figure 1:**
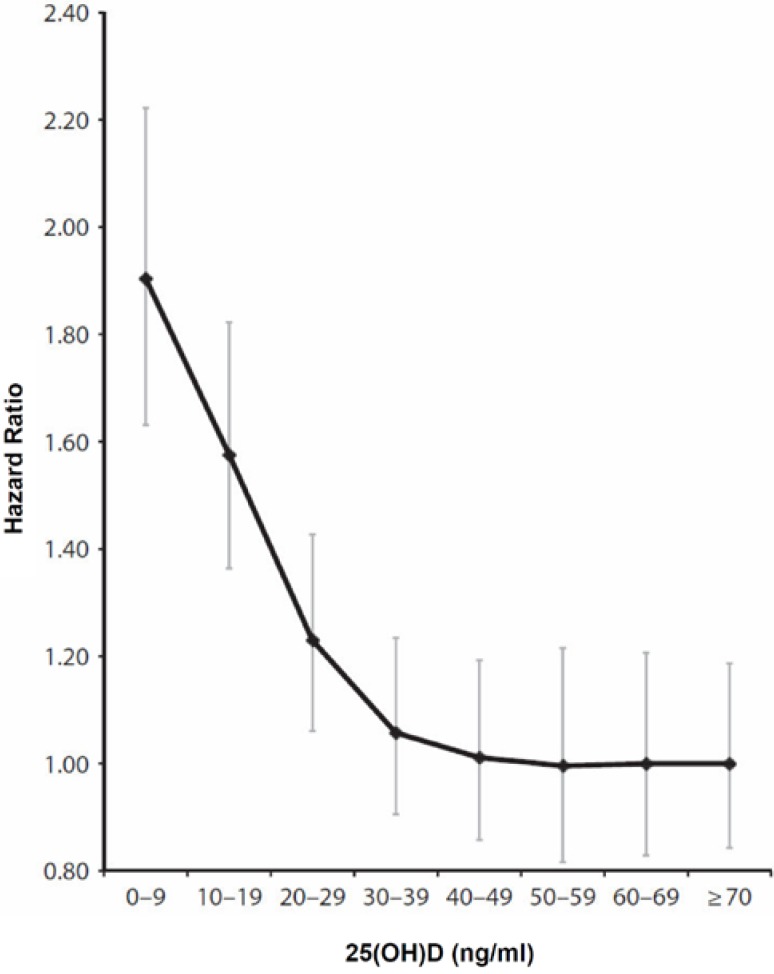
Age-adjusted hazard ratios for mortality of serum 25-hydroxyvitamin D in association with all-cause mortality (reproduced with permission) [19].

The connection between serum 25-hydroxyvitamin D (25(OH)D) and all-cause mortality was re-investigated in a meta-analysis from Garland *et al.* published in June 2014 [19]. This covered a total of 32 studies from January 1966 to January 2013 with more than 500,000 participants (age: approx. 55 years). In 25 of the 32 studies there was a significant relationship between higher serum 25(OH)D levels and reduced all-cause mortality. The results of this meta-analysis provide further confirmation of an inverse relationship between serum 25(OH D levels and the age-adjusted all-cause mortality rate. Individuals with vitamin D deficiency (25(OH)D: 0 to 9 ng/mL, (0 to 22.5 nmol/L)) had a 90% higher risk (hazard ratio 1.9; 95% CI = 1.6, 2.2; *p* < 0.001) of dying prematurely than those with a normal 25(OH)D status (25(OH)D: >30 ng/mL, (>75 nmol/L)) (Figure 1). According to a decreasing exponential curve fit to the data from all studies combined, the point at which the estimated hazard ratio was no longer significantly different from 1.0 was a serum 25(OH)D level of 36 ng/mL (90 nmol/L). In general, serum 25(OH)D levels ≤30 ng/mL (75 nmol/L) were associated with a significantly increased all-cause mortality compared to levels >30 ng/mL (*p* < 0.01). Garland and colleagues emphasized in their paper that the 25(OH)D cut off level for vitamin D deficiency of 20 ng/mL (50 nmol/L) recommended by the US Institute of Medicine (IOM) was too low to make full use of the preventive health effects of the sunshine vitamin with regard to all-cause mortality and the risk of a variety of diseases (e.g., cancer, autoimmune diseases, *etc.*). The cut off level for the 25(OH)D status should therefore not be set at 20 ng/mL, but at 30 ng/mL [19].

Of great concern raised by the Institute of Medicine report was the suggestion that the mortality curve for blood levels of 25(OH)D was U shaped, *i.e.*, there was a decrease in mortality risk with increasing blood levels of 25(OH)D until a level of approximately 30 ng/mL when there was a slight increase in mortality risk [20]. This suggested that there was a narrow window where by improvement in vitamin D status reduced mortality risk and that this benefit was lost when 25(OH)D began to rise above 30 ng/mL. However two recent meta-analyses [19,21] suggests that improvement in serum 25(OH)D to at least 36 and 70 ng/mL was associated with maintenance of reduced risk for mortality. Vitamin D supplementation with 2000 IU to 4000 IU vitamin D3 per day will increase serum 25(OH)D levels above 30 ng/mL [22,23]. It is worth noting in this context that the Institute of Medicine recommended a daily tolerable upper intake level (UL) for vitamin D for persons of nine years and older of 4000 IU and the Endocrine Society recommended for adults an UL of 10,000 IU vitamin D. Until results from randomized clinical trials are available, the researchers consider it reasonable to improve the vitamin D status of the US population by the supplementation of at least 1000 IU vitamin D daily [19]. But recent studies indicate, that 1000 IUs of vitamin D daily especially during the winter time and also in overweight and obese individuals will not raise most adults 25(OH)D blood level above 30 ng/mL (75 nmol/L) [22,23]. Therefore, adults of all racial groups >18 years should follow the Endocrine Society’s recommendations for the prevention of vitamin D deficiency with a daily supplementation of 1500–2000 IU. There is no difference in recommendations to treat or prevent vitamin D deficiency for whites and blacks. They respond to vitamin D in a similar manner and they do not require more vitamin D [22]. Furthermore obese children and adults and children and adults on medications such as anticonvulsants, glucocorticoids or AIDS medications need at least two to three times more vitamin D for their age group to satisfy their body’s vitamin D requirement [22,23,24,25,26,27,28]. As the major source of vitamin D is exposure to natural sunlight, people with a naturally dark skin tone have natural sun protection. Therefore they are at high risk for vitamin D deficiency (25(OH)D < 20 ng/mL). Individuals with a naturally dark skin tone require at least three to five times longer sun exposure to make the same amount of vitamin D as a person with a white skin tone. Thus, we recommend using the serum circulating 25(OH)D level, measured by a reliable assay, to evaluate vitamin D status in individuals which are at high risk to vitamin D deficiency (e.g., Hispanic/Mexican individuals, non-Hispanic black individuals) [22,29,30].

**Figure 2:**
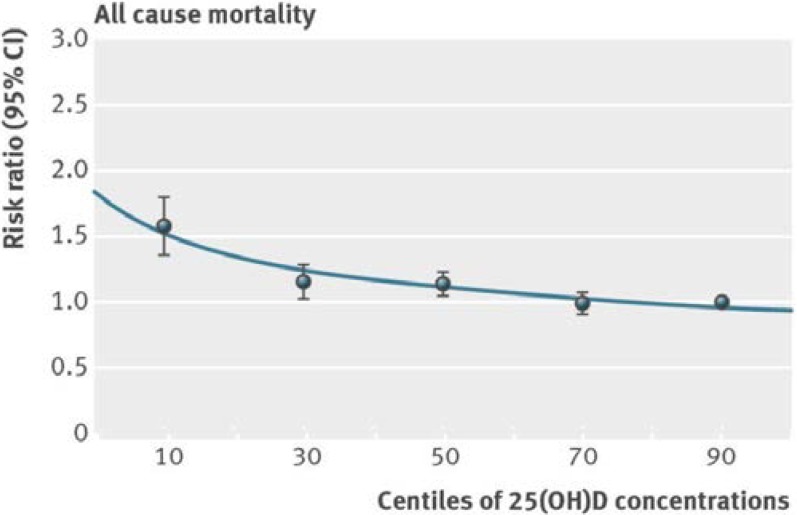
All-cause mortality is higher with low 25(OH)D serum levels (≤10 nmol/L) [21].

A significant inverse relationship between serum 25(OH)D level and overall mortality risk was also observed in another up-to-date meta-analysis that was carried out by the German Cancer Research Centre in Heidelberg. This investigation is a meta-analysis of serum 25(OH)D levels in individual participant data of seven population based cohorts from the Consortium on Health and Ageing: Network of Cohorts in Europe and the United States (CHANCES, www.chancesfp7.eu) together with the third US National Health And Nutrition Examination Survey (NHANES III). The meta-analysis evaluated eight prospective cohort studies from Europe and the USA with over 26,000 men and women (age: 50 to 79 years). A consistent finding across all the individual studies was that the overall mortality of subjects with the lowest 25(OH)D levels ≤4 ng/mL (≤10 nmol/L) was significantly higher (1.57-fold) than in those with a 25(OH)D status ≥36 ng/mL (90 nmol/L) (pooled risk ratio: 1.57; 95% CI 1.36 to 1.81) (see Figure 2). In addition, a separate analysis of cancer mortality showed that cancer patients with a 25(OH)D ≤4ng/mL (≤10 nmol/L) had a 1.7-fold increased risk of dying from the disease compared to cancer patients with a 25(OH)D ≥36 ng/mL (≥90 nmol/L) (risk ratio: 1.70; 95% CI 1.00 to 2.88) [21].

Despite these results, the researchers of the German Cancer Research Centre do not recommend individuals with low 25(OH)D levels to take vitamin D supplements. In the original press release concerning the study of Schöttker *et al.* [21] the German Cancer Research Center recommended until confirmed findings about vitamin D ingestion are available, that in summer time “one should fuel up with the right amount of sunshine, ideally combined with sport and outdoor activities. This would be the way that everyone can ensure an adequate supply of vitamin D and lay down a store for the winter” [31].

It is unrealistic to believe that outdoor activities in the summertime will be able to raise serum 25(OH)D levels to such a significant extent that can be sustained throughout the winter. Typically serum levels of 25(OH)D increase by approximately 10–20 ng/mL by the end of the summer in white Europeans into the range of 35 ng/mL for those exposed to upwards of 300 hours of sunshine per month [32]. Since the half-life for 25(OH)D is approximately 2–3 weeks serum levels decline below the desired 30 ng/mL within 1–2 months after October when sunlight can no longer produce any vitamin D in the skin for those living above 34° North latitude [1,2,3,4]. The lack of any recommendation from the German Cancer Research Centre regarding vitamin D supplementation for cancer patients is regrettable. An association between low 25(OH)D status and increased cancer mortality for colon and breast cancer patients has been documented in several meta-analyses [33,34,35,36,37,38].

## 3. Is It All in the Genes?

Vitamin D deficiency can be due to insufficient production in the skin from sun exposure or to inadequate dietary intake. In addition, there are also may be a genetic predisposition. A Danish study showed that certain genetic variants correlate with a low vitamin D status. The University Hospital in Copenhagen investigated 96,000 Danes possessing the genetic variants associated with 25(OH)D levels in three cohorts (Copenhagen City Heart Study, Copenhagen General Population Study and Copenhagen Ischemic Heart Disease Study). They were observed for several years and the primary endpoint was overall mortality, cancer mortality and other mortalities. The investigators reported that genes associated with low 25(OH)D levels <8 ng/mL (<20 nmol/L) caused a 30% higher mortality risk and a 40% higher risk of cancer deaths. No correlation could be found with the risk of fatal cardiovascular diseases. Genetic variants in the alleles for 7-dehydrocholesterol-reductase (DHCR7) and vitamin D-25-hydroxylase CYP2R1 appeared to be the cause. Although there are several significant limitations that the authors acknowledged regarding their study they did conclude that genetic variance in alleles for 7-dehydrocholesterol-reductase (DHCR7) and vitamin D-25-hydroxylase CYP2R1 appeared to be associated with circulating levels of 25(OH)D [39,40,41].

## 4. Conclusions

Closer attention should be paid to vitamin D deficiency in medical and pharmaceutical practice than has been the case hitherto. The data available to date on vitamin D from experimental, ecological, case-control, retrospective and prospective observational studies, meta-analysis as well as smaller intervention studies, are significant and confirm the sunshine vitamin’s essential role in a variety of physiological and preventative functions [1,2,3,4,22,33,34,35,36,37,38]. The results of these studies are consistent with the recommendation to improve the general vitamin D status in children and adults by means of a healthy approach to sunlight exposure, consumption of foods containing vitamin D and supplementation with vitamin D preparations.

The IOM which used a population model for its vitamin D recommended daily allowances to satisfy 97.5% of the United States population made several assumptions when making their recommendations. They assumed that most children and adults were receiving enough vitamin D from dietary sources and sunlight. They acknowledged the importance of vitamin D for skeletal health and ignored all association studies and ecological studies regarding other health benefits of vitamin D. They also raised concerns that increasing vitamin D intake would increase risk for potential vitamin D toxicity in the general population. However careful scrutiny and further analyses of their data and more recent data has challenged their recommendations. One of the studies that the IOM used in recommending that a blood level of 25(OH)D only needed to be at least 20 ng/mL for maximum bone health is the study of Priemel *et al.* [42] who evaluated 675 German adults who died prematurely in an accident and had their blood recovered for a 25(OH)D analysis and bones for evidence of osteomalacia a hallmark for vitamin D deficiency bone disease. Although the authors concluded that there was no evidence of osteomalacia in these adults who had a serum 25(OH)D >30 ng/mL. However when the IOM reviewed this study they concluded that more than 99% of otherwise healthy German adults ages 20–90 years had no evidence of vitamin D deficiency bone disease osteomalacia with blood levels were above 20 ng/mL and therefore concluded that this level was adequate to prevent vitamin D deficiency bone disease. The IOM however made in error in their evaluation whereby they took the total number of adults who had a blood level of 25(OH)D between 21–29 ng/mL and who had evidence of osteomalacia as numerator and divided it by all adults who had with a blood level of 25(OH)D <29 ng/mL and concluded it was less than 1%. The Endocrine Society [22] pointed out however that the correct method was to take the total number who had osteomalacia with blood levels of 25(OH)D between 21–29 ng/mL and divide by the total number of adults who had this same blood level range of 21–29 ng/mL. When you do this 24% of otherwise healthy German adults who had a blood level of 21–29 ng/mL had evidence of vitamin D deficiency bone disease osteomalacia. Veugelers and Ekwaru [26] concluded that the IOM underestimated the amount of vitamin D required to achieve a 25(OH)D of 20 ng/mL because they made a statistical error in the estimation of the recommended dietary allowance for vitamin D. Ekwaru *et al.* [23] reported that adults taking up to 20,000 IUs daily for more than a year was not associated with any untoward toxicity and their blood levels of 25(OH)D were <100 ng/mL which is considered by the IOM, Endocrine Society and many reference laboratories to be the upper limit of normal. This study also concluded that obese individuals needed 2.5 times more vitamin D to raise the blood levels of 25(OH)D to the same degree as a normal weight person. The Endocrine Society used a medical model for their recommendations for the prevention and treatment of vitamin D deficiency. The recommendations by the Endocrine Society’s practice guidelines of 400–1000 IUs, 600–1000 IUs and 1500–2000 IUs of vitamin D daily for children 0–1 year, 1–18 years, 19–70 years and 70+ years respectively are reasonable daily supplement recommendations to help sustain serum levels of 25(OH)D above 30 ng/mL with the preferred range being 40–60 ng/mL [22]. But there are still disagreements concerning the healthy 25(OH)D level. Furthermore it is important that with the recommendation of vitamin D supplementation the calcium factor should be considered. At this time it is uncertain as to how high blood levels of 25(OH)D have to reach before causing negative health consequences. The Endocrine Society, IOM and many reference laboratories have suggested that the upper limit of normal for 25(OH)D be 100 ng/mL. This recommendation is solely based on the calcemic effect of vitamin D and not on other health consequences including mortality. Further investigation is required to better understand what blood level of 25(OH)D is required for maximum health [20,22,43,44,45].

Indeed Grant [46] suggested that a significant reduction in mortality rates could be achieved by doubling vitamin D levels. Besides prospective studies on how to assess vitamin D deficiency in this special setting, it will be crucial to perform large, well planned multicenter randomized controlled trials to evaluate the effects of vitamin D on clinical outcomes, taking into account the important basic rules regarding the design for clinical studies on nutrient effects [47]. There has been a suggestion that the reason for the association between low 25(OH)D and many chronic illnesses is due to the fact that the inflammation associated with chronic illnesses was causing the vitamin D deficiency [48]. This has been challenged by Holick and Grant [49] who noted that the authors of the study misinterpreted the literature supporting their hypothesis.
